# Mapping social movements momentum: unveiling networks in the movement for the right to abortion in Mexico

**DOI:** 10.3389/frma.2024.1294495

**Published:** 2024-05-24

**Authors:** Cesángari López Martínez, Allison Sambo, Diana Medina González

**Affiliations:** ^1^Global Fund for Women, San Francisco, CA, United States; ^2^Fondo Semillas, Mexico City, Mexico

**Keywords:** abortion, intersectional feminisms, movement mapping, Mexico, social network analysis, social movements, surveys

## Abstract

In this case study, we propose a network analysis approach to map social movements through an intersectional feminist lens. We aim to gain a more in-depth understanding of movements' network structures to examine the roles and relationships of movement actors, the flow of resources between them, and potential areas of collaboration and conflict. By incorporating Social Network Analysis (SNA) techniques and visually mapping dynamics within social movements, this approach can assess the significance of small actors in creating change and stresses the need for their perspectives to be heard. Furthermore, our methodology adopts a feminist intersectional framework that recognizes the role of different identities in shaping the movement and its actors. To demonstrate the practical application of this approach, we examined the movement for the right to abortion in Mexico between September and December 2021. Our multi-step process included (1) designing a survey tool adapted to the context of the movement, (2) collecting and analyzing survey responses on movement actors' relationships and interactions, including their priorities, activities, needs, risks, and challenges, (3) visualizing the network using SNA techniques that account for the complexity and diversity of the movement, (4) interpreting the findings through insights collected in semi-structured interviews and validation meetings with key movement actors, and (5) implementing safety and security guidelines to safeguard the identity of individuals whose activities could put them at risk of suffering institutional, emotional, and physical violence. Our case study offers valuable insights that not only encourage the integration of feminist and intersectional perspectives into data collection processes but also provide a roadmap for accompanying social movements and supporting meaningful and contextually responsive activities.

## 1 Introduction

As social movements endeavor to disrupt existing structures and foster collaboration, it becomes crucial for movement actors to comprehend their potential for coordinating and advancing their shared visions. To achieve this understanding, it is essential to examine the network dynamics and relational resources at play within their networks. Drawing on the extensive literature on social movements, we emphasize the profound significance of interactions and resource exchange among social movement actors, highlighting both the challenges and opportunities inherent in collaborative activities toward common goals (Diani, 1992, 2003; Tarrow, 2001). We present a mapping methodology guided by an intersectional feminist perspective that acknowledges variations in movement actors' influence and decisions based on their roles within the movements, gender, race, age, and other identities. Our approach utilizes Social Network Analysis (SNA) to map relationships within social movements, identifying network structures and examining opportunities and challenges to collaboration within them. Furthermore, we argue that our proposed approach can play a pivotal role in informing strategies related to funding, resource allocation, and networking, particularly in situations marked by disparities in access to resources and influence in decision-making. This perspective enables a more nuanced analysis of how movements can be strengthened and sustained.

To demonstrate the practical application of our mapping methodology, we conducted a multi-step, mixed-methods case study, integrating surveys, SNA techniques, semi-structured interviews, and validation exercises to visualize the networking structures of a social movement. Our case study focused on the movement for the right to abortion in Mexico, covering the period from September to December 2021. Through this analysis, we gained insights into movement actors' relationships and interactions within the movement, identifying key players, their roles, the flow of resources, and sub-networks.

Our methodological approach and the findings resulting from its implementation in the case study yielded an understanding of the dynamics and accomplishments of the movement for the right to abortion in Mexico. Notably, our study also shed light on emerging sub-networks and movement actors who may have been overlooked, challenging mainstream perceptions regarding their impact and the most urgent priorities and struggles experienced in the movement during the analyzed period. We believe that by acknowledging and addressing these dynamics, social movements can strive to amplify marginalized voices, incorporate diverse perspectives, and foster participatory decision-making processes. This understanding is a foundation for developing strategies to accompany grassroots organizations' journeys, promote intersectional approaches, and create inclusive spaces for underrepresented groups.

In the following sections, we provide a detailed explanation of our proposed movement mapping approach. First, we outline the social movement literature that guided the design and implementation of our methodology. Then, we discuss the primary goals of our mapping approach, the collection of survey data, the mixed-methods analytical techniques utilized, and the measures taken to ensure the privacy and security of participants' information. Subsequently, we present the findings derived from our case study, highlighting the unique characteristics of the movement, the diverse roles and resources of movement actors, and the complex structures and sub-networks within the movement. Furthermore, we discuss the relational dynamics identified within the case study, examining how influence and decision-making are distributed among participants. Finally, we conclude with the key insights gained from the case study and reflect on the limitations encountered while implementing the proposed methodology.

## 2 Literature review

### 2.1 Social movements as networks of relationships

The study of social movements aims to differentiate them from other types of collective action, highlighting their complex and evolving network structures (Diani and McAdam, 2003). Scholars such as Diani (1992) and Tarrow (2001) emphasize that social movements are best understood as networks of interactions between various actors, including formal and informal organizations engaged in prolonged and potentially confrontational interactions (Moyer et al., 2001). Networks and relationships are crucial in defining social movements, encompassing infrastructure, ideologies, and formal and informal relationships between movement actors (Diani and McAdam, 2003; Edwards and McCarthy, 2004a).

Studying social movements as networks means looking at how actors, organizations, and events connect. This evidences how they depend on each other, coordinate, experience social conflict, share identity, maintain individuality, and exchange resources within informal networks (Diani and McAdam, 2003; Edwards and McCarthy, 2004b). Further, analyzing social movements as networks enables network analysis, whereby the concentration, dispersion, or lack of collaboration and access to resources can be described and potentially explained. Utilizing these insights, our network analysis methodology focuses on understanding the presence, position, and intermediation of actors within the movement network, emphasizing the importance of interactions and resource exchange among them. Our framework underscores the significance and methodological depth added by SNA in visualizing positionality and relational structures, particularly centrality, among movement actors^1^.

### 2.2 The flow of resources within movements

Understanding the dynamics of resource flow within social movements is crucial from a relational perspective, shedding light on collaborative efforts and the quality of interactions among individuals and organizations (Edwards, 2014). Resource theories highlight the significance of actor characteristics and relationships in the functioning of social movements, while social capital theories emphasize the role of relationships as resources for specific actors and movement goals. Social capital is critical in mobilizing a strong base, bridging alignments with those indirectly affected but aligned and accessing power holders for lobbying and resource acquisition (Fayong, 2008). Social capital also implies degrees of power to influence the direction and strategies of the movement through different types of relationships.

Social movements encompass a range of resources that collectively contribute to their functioning and effectiveness. Edwards and McCarthy (2004a) categorized social movement resources into several key types. Moral resources, such as public recognition, popularity, and external support, are bestowed upon the movement. Cultural resources involve established ways of operating within the movement, including tactics, approaches, and technical know-how aligned with the movement's vision and culture. Socio-organizational resources refer to infrastructure, social networks, and supporting organizations aiding mobilization efforts. Human resources extend beyond labor and skills, encompassing the effective utilization of these resources. Material resources include financial and physical assets available to the movement, such as grants, donations, office spaces, and equipment.

A considerable amount of scholarship examining social networks, including personal and professional connections, explores their role in mobilization. Relationships (both direct and indirect connections) among collaborating organizations can be leveraged for mobilization. This underscores the importance of preexisting ties within social movements. For example, the interconnectedness of individuals in formal, informal, and virtual networks, facilitated by social capital and communication channels, shapes the mobilization potential of a movement (Klandermans, 2013). And activists utilize their personal and professional social networks for ongoing mobilization, a phenomenon known as “bloc recruitment” (Minkoff, 1997; Diani and McAdam, 2003; Edwards and McCarthy, 2004a,b).

Beyond resources for mobilization, individual organizations can exchange, leverage, or co-produce resources through relationships. Relationships can be considered direct or indirect and can be evaluated in terms of social capital by bonding amongst similar individuals or organizations, bridging amongst dissimilar individuals or organizations, or linking to outside and potentially allied groups (Diani and McAdam, 2003; Fayong, 2008; Nowell, 2009; Kropczynski and Nah, 2010; Klandermans, 2013). A movement's structure, functioning, and available resources are shaped by the number and type of relationships within its network (Diani, 2003). Strong ties among a smaller cohort foster consolidated networks with higher trust and more sharing, while weak ties enable diversity, growth, and exploration of new opportunities (Sommerfeldt and Yang, 2017). This implies no ideal in terms of network structure, but rather functional aspects of different structures produced through relationships.

### 2.3 Actors' positionality and network structures

In the realm of social movement literature, insights from seminal works on social network analysis contribute significantly to understanding movement actors' positionality and intermediation within a social network. The exploration of centrality by Freeman (2002) establishes a foundational understanding of key measures like degree centrality, betweenness centrality, and closeness centrality. This conceptual clarity aids in identifying those central actors with a high number of relationships (degree centrality), emphasizing their structural importance within a movement.

Moreover, Freeman's insights into betweenness centrality contribute to delineating how certain actors function as intermediaries, shaping communication and resource flow within the network. Similarly, Wasserman and Faust (1994), further enrich the literature by covering various centrality measures in identifying nodes that act as bridges or intermediaries between others. This literature, complemented by Scott (2012), underscores the significance of degree and betweenness centralities as key measures for identifying intermediaries and brokers, providing valuable insights into the dynamics of information and resource exchange within social movements.

Network structures within social movements must also be analyzed to gain insights into movement composition, relationship qualities amongst actors, density of resources, and identification of key actors for specific objectives. These structures provide valuable information about how coordination occurs and whether the networks function as movement processes, coalitions, organizations, or other forms of coordination. Sommerfeldt and Yang (2017) show that different kinds of network ties are important to different stages, and social movement organizations should build or strengthen either a diversity, quantity, or type of tie at different points. For example, in the initial stages of a social movement, having diffuse networks with numerous informal connections could be advantageous for sharing information and establishing broad support. However, as movements progress, having fewer but stronger ties or a smaller number of actors with denser connections can facilitate more coordinated, strategic, and swift actions.

Diani (2003) underscores the significance of understanding the role of networks in social movements, emphasizing the exchange and possession of resources within these relationships and their support for collective action. Mapping tools, according to Diani, have the potential to inform and guide action by analyzing social movement networks in terms of centrality and segmentation. This involves scrutinizing metrics such as ties, qualities, and direction to comprehend actor dynamics and the dissemination of connections. Diani's research also illustrates how the structure of relationships within a social movement significantly influences its functioning, identifying key actors and emphasizing that there is no one-size-fits-all structure. Instead, different network structures provide insights into the diverse roles of movement actors.

As highlighted by Diani (2003), the different network structures have their strengths and weaknesses. For instance, a “Clique” structure promotes equal information sharing and inclusive decision-making but may face challenges of excluding new members and encountering delays in quick action due to information flow through all actors. On the other hand, a “Wheel” structure heavily relies on a central actor, risking overdependence and potential dysfunction if that individual is unavailable. “Segmented” networks exhibit strong relationships within sub-networks but lack integration across them, while “polycephalous” networks employ brokers to link centralized sub-networks, common in movements consisting of coalitions or campaigns.

Crucially, actors also play a pivotal role in connecting isolated factions or groups, even if they are not centrally positioned. Chaudhary and Warner (2015) classify these actors, known as brokers, based on their diverse roles in facilitating connections and resource distribution within social movements. These roles include liaison brokers, bridging two groups without fully belonging to either; itinerant brokers, serving as “consultant brokers” within the same group; coordinator brokers, operating within a single group and facilitating communication among its actors; gatekeeper brokers, establishing connections with external actors and controlling the flow of information and resources to their group; and representative brokers, representing their group in negotiations with external entities and managing the exchange of information and resources in a distinctive manner.

### 2.4 NSM and women's and feminist movements in Latin America

The developed and piloted movement mapping process wove three strands of the literature on social movements and social networks together in a practical application. First, social movements are complex networks comprising both formal and informal relationships among organizations, individuals, and events. Second, these relationships' presence, absence, and structure are crucial in shaping the accumulation, sharing, exchange, and generation of resources within movements. Third, actors' positionality and role and network structures can be useful in describing movement qualities, identifying critical bridges or inhibitors of information flow, and documenting coordination processes.

Additionally, we defined the case study parameters and designed qualitative data collection instruments within the framework of New Social Movement (NSM) theories. This framework guides our understanding of women's and feminist movements in Latin America, emphasizing insights into resource mobilization, contextual impact, the significance of identities, and movements' relevance and impact beyond their geographical borders (Alvarez, 1990). By incorporating a comprehensive understanding of NSM theories with threads of resource mobilization, political processes, cultural dynamics and global collaboration, we developed a theory-backed, contextualized survey and interview mapping process. This approach is pivotal in unraveling the complex tapestry of feminist and women's movements in Latin America, providing a nuanced and powerful lens through which their successes and challenges can be fully appreciated.

Through the lens of NSM theories, particularly Resource Mobilization and Political Process Synthesis as articulated by Tarrow (1996), we highlight the pivotal role of resource mobilization and the political context in shaping the successes and challenges faced by feminist and women activists. Cultural theory further enriches our understanding by emphasizing the significance of identity, symbolism, and cultural narratives within feminist and women's movements across the region, as elucidated by Jaquette (1989). Identity politics and values-based approaches, as outlined by Fraser (2020), underscore that these movements inherently revolve around critical issues such as gender, sexuality, and reproductive rights. Their overarching goal is to secure recognition and justice for women and marginalized gender identities. Moreover, exploring the transnational and global dimensions of these movements is imperative, and this is accomplished through the application of globalization and network theory, as conceptualized by Alvarez (1990). This perspective underscores the movements' remarkable ability to transcend borders and collaborate on issues that resonate across diverse societies.

These foundational principles naturally facilitate an exploration of the dynamic interplay within relationships in social movements, revealing how specific actors and their collaborative efforts gain leverage in accessing diverse resources. This seamless alignment with our investigation into the dynamics of movement actors and their relational interactions underscores the significance of mapping as a pertinent and effective analytical tool. This approach not only emphasizes the relevance of our case study but also provides valuable insights into how movements strategically prioritize issues, formulate strategies, and take action in pursuit of collective goals.

## 3 Materials and methods

### 3.1 Purpose of the movement mapping process

This section presents our approach to social movement mapping. This methodology was developed through a collaborative effort between Global Fund for Women^2^ and a cohort of feminist and women's funds^3^. The purpose of the methodology is to comprehensively describe the composition, structure, and functions within social movements by examining movement actors and their relationships. Through this descriptive exercise, the methodology provides insights into how resources and movement actors' interactions can shape the movement's actions. Our ultimate goal is to offer actionable recommendations for movement actors, allies, and funders to accompany unheard voices by providing financial and non-financial resources to support their activities.

Our mapping analysis focuses on movement characteristics, actors' roles and resources, and movement structures and sub-networks. These three domains provide valuable information for discussing the network's demographic composition and dynamics, including which actors are connecting with each other, the priorities they are focusing on within the movement and across demographic groups, potential reasons for disconnects, and challenges to collaboration. The design and implementation of the case study resulted from a collaboration between the learning and evaluation teams of Global Fund for Women and Fondo Semillas^4^. This methodology section outlines our process, focusing on analyzing individual and movement-level resources and the form in which they flow within the network to gain insights into how these factors shape movements' actions.

In what follows, we outline the two-part, interwoven data collection process that combines a survey designed to gather information on individual actors (affiliated and not affiliated to a group) and their relationships within the movement network with semi-structured interviews and data-validation exercises to further the understanding of survey findings. The mixed-methods analysis strategy is also presented, including key metrics, their interpretation, and their application in analyzing movement dynamics and promoting action. Lastly, we discuss the privacy and security practices implemented to protect participants' sensitive information.

### 3.2 Survey design

Our methodology's primary data collection method is a survey tool, enabling systematic information collection for analyzing and visualizing social movement networks. Building on Global Fund for Women's Movement Capacity Assessment Tool (MCAT)^5^, the survey questionnaire serves four distinct purposes: identifying movement actors and relationships, exploring actors' roles, examining the resources actors provide or need (including capacity dimensions, priorities, and strategies), and investigating the overall network structure and sub-networks or communities of interest. The questionnaire comprises four sections: demographic information, respondents' affiliation and role in the movement, movement capacity assessment (MCAT questions), and network characteristics and snowballing.

The survey sections collectively provide comprehensive data on social movements and their networks. The demographic information section captures background details about the respondents, contextualizing their involvement in the movement. The section on respondents' affiliations and roles identifies their contributions to advancing the movement's objectives. The movement capacity assessment section evaluates the movement's overall strength and focus areas, shedding light on its resources, challenges, and potential growth strategies. Lastly, the section on network characteristics and snowballing examines the structure of the overall network and the relationships among movement actors, organizations, and communities of interest while also implementing a snowballing mechanism that requests respondents to refer other potential participants, especially those identified as their most significant relationships within the movement, who can contribute valuable insights.

Each section plays a vital role in mapping respondents along with their demographic profiles. This mapping is facilitated by connecting each respondent with individuals they have self-identified as their primary connections within the movement. This approach unveils the intricate web of connections, allowing us to comprehend affiliations and roles. These insights serve as a foundation for subsequent discussion and sense-making during interviews and validation meetings.

Table 1 illustrates an example of a movement actor profile created for each survey respondent – in this case, a female who provides abortion accompaniment services and reports connections with at least three different movement actors for capacity-strengthening activities and the exchange of financial resources and information. As described in a subsequent section, the left part of the profile, which pertains to self-reported demographic characteristics and movement perceptions, was available for 225 respondents, while the right part, covering actors' connections within the network, was available for 176 respondents.

**Table 1:** Example of a movement actor profile.

**Demographics and movement perceptions**			**Connection 1**	**Connection 2**	**Connection 3**
Age: 44	Gender: Female	Location: Chihuahua	Organization	Organization	Individual activist
Main focus: Comprehensive sexuality education			Connects twice a year	Connects twice a year	Connects once a year
Main role: Healer providing abortion accompaniment services Main contribution: Self and collective care			Connects to receive capacity strengthening	Connects to exchange financial resources	Connects to exchange information
Main movement need: Leadership development Movement stage: Emerging			On a scale from very unlikely to very likely, she responded to be very likely to do the following activities with each of the reported connections: a) exchange financial resources, b) collaborate in joint activities, c) share confidential information, and d) refer them to other movement actors		
Three main affiliations: one formal organization and two national networks of groups providing abortion accompaniment services					

The survey questionnaire designed for the case study comprised 22 core questions and had an estimated duration of 20 to 30 min. The duration varied depending on the number of connections described by participants – participants had the option of detailing up to three of their most important connections within the movement at the moment of data collection. The questionnaire was adapted and translated into Spanish by the Fondo Semillas team to ensure a context-responsive translation. The survey questionnaire is available in Appendix 1^6^.

All survey questions used to create the movement actors' profiles and connections were presented as multiple-choice or Likert scale, requiring participants to select only the options that best described their situation. The response options were tailored to the movement, following common social movement concepts and categories already proven in other movement assessment processes such as the MCAT. The questionnaire also featured a set of open-ended questions employed by the analysis team to steer the interviews, pinpoint topics omitted by the questionnaire and response options, and gather feedback for the case study teams. The data collection process was facilitated through Qualtrics, with survey invitations and sample snowballing conducted using the same platform^7^.

### 3.3 Survey collection and response processing

We distributed the survey to over 300 contacts compiled by the Fondo Semillas team, including grantee partners, peers, allies, and personal connections. A snowballing mechanism was employed to ensure a broader and more diverse sample of potential movement actors, resulting in over 400 survey invitations being sent. The survey remained open from September 6 to November 15, 2021, with 225 individuals responding. The respondents represented all 32 states in Mexico, with a median age of 38 years. The majority identified as female (91%), while a smaller percentage identified as male (2%) or non-binary (2%). Geographically, the most represented states were Mexico City (11%), Puebla (8%), Yucatán (6%), Baja California (6%), Guanajuato (5%), Quintana Roo (5%), and Chiapas (4%). Notably, 35 respondents (16%) reported a presence in multiple states, and it's important to clarify that the most represented states are not necessarily close to each other geographically. This indicates that responses were distributed across the country, with at least three respondents (1%) from each state.

A critical step in the information processing was determining which responses would be used for the analysis of the overall perception and which contained sufficient information for the mapping of the network. Some respondents, possibly due to survey fatigue according to feedback gathered through the process, only completed the initial part of the questionnaire without providing information on their relationships. Additionally, we encountered duplicates, highly incomplete responses, and test responses. Our criteria aimed to retain as many unique responses as possible, ensuring a comprehensive view of respondents' perspectives on the movement and their relationships within it.

All 225 respondents provided information regarding their perception of the movement, and 176 (78%) had sufficient information to map relationships^8^. These 176 responses allowed us to identify 350 unique movement actors (including respondents and their relationships) associated with 469 relationships. Forty-six relationships had to be excluded from the mapping of the network due to issues related to data quality and completeness. Despite these challenges, we successfully mapped and characterized 423 relationships, indicating that, on average, respondents identified 2.4 relationships.

Cleaning the survey responses involved an intensive manual assessment, including correcting typos and misspellings and matching acronyms with incomplete or complete names of groups. Data cleaning was led by the Global Fund for Women team, with ongoing consultation of Fondo Semillas staff to ensure consistency in interpretation and accuracy. This process included defining whether entities such as “Global Fund for Women,” “GFW,” and “global fund for women” corresponded to the same group. When it was challenging to clean individual names, efforts were made to replace them with the names of their affiliations. Moreover, when relationships reported by respondents involved names of individuals who did not respond to the survey (i.e., did not provide consent to use their individual name), we either anonymized them (e.g., Respondent 1) or substituted them with the names of their respective groups when available. The Fondo Semillas team utilized their internal directories and contextual knowledge to the fullest extent possible to match responses.

While we assert the absence of a conflict of interest, as the primary objective of this study is to provide a roadmap for accompanying social movements and supporting meaningful and contextually responsive activities, it is important to acknowledge the influence and potential overrepresentation of Fondo Semillas' perspectives in our mapping process and findings. These concerns emanate from the integral role played by Fondo Semillas in compiling the sampling frame, implementing the methodology and developing this manuscript, coupled with their established influence and stature within the women's and feminist movements in Mexico. Nevertheless, it is noteworthy that the final composition of the response universe included 188 responses (84%) with no direct affiliations or direct relationship with Fondo Semillas^9^.

### 3.4 Mixed-methods analytical approach

Our mixed-methods analysis involved transforming our survey-collected information into visual data and validating survey findings through semi-structured interviews and validation meetings. The survey gathered information about connections among movement actors, including individuals and organizations, and measured their attributes, such as organizational affiliation, role in the movement, and perceptions of movement needs, priorities, and challenges. So, to effectively map the social movement, we applied SNA techniques and characterized actors based on these self-reported characteristics. This enabled us to visualize network structures and patterns of social relationships, ultimately identifying key actors and dynamics within the movement. This included assessing trust, alignment, and types of exchange in relationships, as well as utilizing centrality measures to identify actors' positionality and intermediation within the network. Key centrality metrics such as degree centrality, closeness centrality, and betweenness centrality were calculated.

To gain a deeper understanding of the survey data, we conducted ten semi-structured interviews and two validation meetings with various movement actors, specifically targeting those with demographic profiles that may have been underrepresented in the SNA analysis. The Fondo Semillas team carefully curated the selection of interviewees to ensure diversity in identities, geographic representation, and modes of participation within the movement. With the support of a local interviewer deeply embedded in the Mexican women's and feminist movements, the Fondo Semillas team conducted the interviews virtually. Simultaneously, they shared updates and findings to inform the analyses of the Global Fund for Women team working on the quantitative calculations. While not all interviewees necessarily participated in the survey or provided complete responses for network mapping, their profiles aligned with the characteristics measured in the survey, even if some were notably underrepresented. The objective in identifying interviewees was to engage with individuals representing all mapped gender identities, diverse geographical locations, and various forms of contributions to the movement. We aimed to ensure at least one representative from each demographic group was interviewed, providing ten converging and diverging perspectives from ten distinct profiles.

The average age of the interviewees was 38, with the youngest participant being 18 years old. Nine interviewees identified as women, while one identified as a man. They resided in various states, including Baja California, Colima, Guanajuato, Michoacán, Mexico City, Nuevo León, Querétaro, Veracruz, and Yucatán. Additionally, some interviewees were involved in work and supported movements in Central America and the United States. We also organized post-analysis validation meetings in Mexico City, inviting participants from the City and other states to provide their reactions to the results and define actions based on them.

Validation meetings were hosted and facilitated by the Fondo Semillas team in Mexico City and were attended in person by over 50 participants each. In the first meeting, participants had the option of engaging with the interactive version of the map of the network and exploring the different movement actors and their characteristics and relationships. In the second meeting, discussions were more focused on preliminary reflections built with insights from the survey, interviews, and the previous validation meeting. It is important to highlight that other topics, not necessarily aligned with the abortion rights movement but involving other feminists and women's movements, were also discussed during the convenings.

Both exercises were motivated by questions related to discussing the implications of the mapping exercise for (a) identifying activities and strategies requiring more effort or resources, (b) promoting collaboration and coordination among movement actors, (c) understanding the demographic differences observed in responses, (d) including excluded groups in movement activities, and (e) addressing specific collaboration challenges. A more detailed description of the discussions is presented in the next section.

Interviews and validation meetings took place from March 2022 through March 2023 and helped us make sense of the survey data and validate the findings, ensuring a comprehensive understanding of the movement and its dynamics. Both interviews and validation meetings scheduling were significantly impacted by COVID-19 travel restrictions and local policies for in-person gatherings.

### 3.5 Privacy and security

Acknowledging the nature of the analyzed movement, the existence of power structures, and the prevalence of violence within the Mexican abortion and reproductive rights context, we followed strict privacy and security guidelines based on consent protocols to safeguard the identity and activities of survey respondents. To ensure ethical practices, we obtained informed consent from all individuals involved before collecting any data. Participants were provided with clear and comprehensive information about the purpose of the study, the data collection methods, and how their data will be used and stored. The consent form was embedded in both the survey invitation email and the welcome message of the survey, generating a two-step process in which participants were twice shown the purpose of the analysis and data and privacy policies before starting the survey. On both occasions, participants were informed that by responding to the survey, they agreed that the personal information provided (names, affiliations, and email addresses) was going to be used for the case study purposes only. The invitation email that was also used as the survey welcome message is presented in Appendix 2.

Consent, as well as data storage, management, and deletion protocols, adhered to an internal data protection procedure reviewed and approved by Global Fund for Women counsel. This included protocols for data collection, storage, access, management, and deletion to protect the identities of individuals. Where possible, automations were made so that potential participants could independently revoke their data. Participants receiving the invitation had the option to remove their names and information by an opt-out button, effectively deleting their information from our databases. Email addresses were briefly retained on an “opt-out” list to ensure the system did not re-populate a message to someone who had chosen to opt out if they were subsequently nominated.

Respondents who clicked on the survey link included in the invitation email were again made aware of the purpose and scope of the research through the welcome message and asked to provide their name and contact information if they consented to participate. Our data privacy policy also interpreted non-response as a request for opting out and similarly deleted the information received, retaining only briefly to ensure no future communications were sent. In the case that an invitation letter was sent, either from our initial contact list or through the nomination (snowballing) process, but not responded to, the name and email address of the individual or group were deleted from our databases. Participants, in both the invitation email and welcome message, were instructed of their rights to withdraw consent at any time with no consequence and provided necessary contact emails to revoke their data.

All data collected was securely stored and accessed only by authorized personnel. We strictly adhere to the principle of storing and using only the data that participants consented to share. Unconsented information, including any personal identifiers, was promptly de-identified to maintain confidentiality. We carefully removed or anonymized any direct or indirect identifying information to ensure the privacy and protection of participants' identities. Network maps and other visualizations supporting the analysis were password-protected, and we only shared anonymized versions with audiences beyond the case study teams.

In accordance with the privacy policy of the General Data Protection Regulation (GDPR), our data storage practices follow the guidelines and principles outlined by the Global Fund for Women's and Fondo Semillas' privacy policies. This includes implementing appropriate technical and organizational measures to prevent unauthorized access, disclosure, or data loss. We handled all data with utmost care and respect for participants' privacy to safeguard the confidentiality and security of the information collected.

## 4 Results

### 4.1 Mapping the movement for the right to abortion in Mexico

This section presents the key reflections from the case study, focusing on the three mapping domains: movement characteristics, actors' roles and resources, and movement structures and sub-networks. Drawing from both survey data and the semi-structured interviews, this section provides an overview of relevant findings demonstrating the scope and advantages of implementing our mapping methodology. Furthermore, it underscores the critical importance of the analyzed movement, which is the movement for the right to abortion in Mexico. The insights garnered from this analysis play a pivotal role in the subsequent section, the Discussion, where we delve into the nature and dynamics of the movement's relationships, aiming to uncover any disparities in actors' experiences, resource access, and influence within the movement.

The presented survey results are included for descriptive purposes, illustrating the type of information that the survey and SNA could retrieve for movement actors' sense-making (interviews and validation meetings). All survey data percentages below were calculated over the complete universe of respondents (*N* = 225) unless specified otherwise. By survey design, participants had the option of skipping questions when the statement did not apply to their context or when they did not feel comfortable responding. Skipped responses were labeled as such and were retained in the calculation of distributions. For a more detailed account of the collected data, readers are referred to the comprehensive report titled “Constellations of the movement for the right to abortion in Mexico,” developed by Fondo Semillas and published in June 2023 (Alcázar et al., 2023)^10^.

### 4.2 Movement characteristics

The case study delved into the movement fighting for the right to abortion in Mexico. This movement is a crucial component of the women's and feminist movements in the country. It unites a wide range of feminists, allies, and individuals who acknowledge the right to individual independence over their bodies and reproductive decisions. The study coincided with a transformative period in the history of the abortion movement throughout the Americas (Marea Verde movement), with significant shifts in Mexico's political landscape and the groundbreaking decision by the Mexican Supreme Court of Justice in early September 2021 to declare the criminalization of abortion unconstitutional^11^.

Survey data revealed that most respondents (76%) were affiliated with at least one group within the movement for the right to abortion, with formal organizations, collectives, and support networks being the most frequently mentioned form of affiliation. Twenty percent responded as individual activists, although this does not imply that they are not affiliated with a group, and the remaining 4% did not respond to the question. Respondents' engagement forms varied based on location, with individuals in Mexico City more involved in formal organizations (46% of those located in the city), while those outside the capital were more engaged with informal support and collective networks (35% of those located outside the city were involved in formal organizations). According to survey respondents, the primary goals of the movement included advocating for the decriminalization of abortion nationwide, legal changes at federal and state levels, and the release of incarcerated women due to abortion^12^. According to interviewees, activities aligned with these objectives included lobbying, engaging with decision-makers, community-based initiatives, workshops, educational/artistic endeavors, media communication, and coordination among movement members.

The survey findings indicated that the movement for the right to abortion in Mexico was perceived by most respondents as “popular” (63%) and “formalized” (21%). Fewer than a fifth of respondents considered the movement in the extremes of its life cycle, either by regarding it as “emerging” (12%) or in “decline” (4%)^13^. According to interviewees, the movement gained visibility through large-scale actions, increased recognition, and alignment among participants. It had also experienced expanding media coverage and greater public engagement and was becoming a significant political force. Interviewees also observed the movement's growth, with more people joining and contributing unique perspectives, emphasizing its continued momentum and positive trajectory. For example, one interviewee, when asked about this finding, mentioned: “Over the years I have seen many changes; everything that had not happened at the level of public opinion and politics is happening now. I find it very important; it has not stopped. [It is] visible, more and more little green handkerchiefs^14^ on the street, and I say to myself, how beautiful, I think that there are more and more people who have taken up the movement and see it from their perspectives.”

### 4.3 Movement actors' roles and resources

The survey results provide insight into the makeup of the movement fighting for the right to abortion in Mexico and how various actors contribute and interact with other members. The majority of those surveyed identified themselves as a part of the abortion and women's movements in general (97%) while some hesitated to associate with the feminist movement due to its perceived lack of intersectionality and racial and class inclusion. Participants' main areas of concentration were sexual and reproductive rights (54%), abortion decriminalization at local levels (16%), and support for medical abortion processes (11%). When specifically asked about their support for abortion accompaniment processes, 64% responded that they had provided some type of support (direct accompaniment, midwife services, healer, health professional, etc.).

Survey respondents reported making diverse contributions to the movement, with common areas of involvement being base-building activities (27%), coordination and collaboration with other movement actors (18%), and contributing to developing a shared vision and narrative (16%). Interviewees reported strong interactions within the movement, as evidenced by a shared and clear vision, effective communication strategies, including affected individuals' voices, and responsive leadership. Additionally, both the results of the surveys and interviews suggest good collaboration and coordination practices, including horizontal listening and creating spaces for sharing diverse experiences and financial and non-financial support.

Findings identified the existence of inclusive meetings and regional gatherings, which interviewees saw as crucial for ensuring the decentralization of the movement's agenda from Mexico City to the rest of the country and from the mainstream organizations located in the capital to other groups and networks working in other states. Also, the collected data suggests a consensus on the need for a more unified national strategy, particularly for current strategies, including agreeing on specific gestational week limits in legislative proposals and changes to local penal codes.

However, survey and interview data also unveiled a clear division among movement participants regarding including transgender individuals in the movement^15^. Interviewees noted that those excluding transgender individuals displayed traits associated with whiteness and upheld conservative perspectives on the broader feminist movement. Conversely, the group advocating for inclusion was perceived as to have adopted an intersectional approach but faced challenges in reaching a unified message. Survey, interview, and validation meetings' participants identified the presence of exclusionary and discriminatory attitudes toward trans individuals as the main obstacle to collaborative efforts among movement participants. This challenge was particularly emphasized by interviewees who reported choosing not to engage with groups and activists who did not recognize trans women as women and feminists.

Notably, certain interviewees described facing transphobia within specific feminist circles as directly impeding the smooth flow of resources on the ground. They pointed out that transphobia takes two main forms. The first is the refusal to acknowledge trans women as integral to the feminist and women's movement, even when groups led by trans women are offering safe abortion services. The second form involves excluding trans men and individuals with the capacity to gestate, with some exclusionary feminists explicitly advocating that abortion rights and access should be restricted to “biological women.” One interviewee expressed this concern through a powerful statement: “There [are] always some differences on issues [within the movement]. But now, there are trans-exclusionary or hating positions in some cases. There are historical referents [that do not] recognize trans women as women or as feminists. I am concerned that these positions deny the identity of other people, and I consider it a ‘deal-breaker' to be able to work with some colleagues. Something that worries me, something that we have discussed, are these organizations that are transphobic, they are in favor of abortion but for ‘biological women,' which is nonsense. We are concerned because in the state, the [people] close to the [political] parties are transphobic.”

Although concerns emerged about the movement's capacity to encompass individuals from diverse socioeconomic backgrounds and various geographic locations, these concerns were identified to a lesser degree. Likewise, a few interviewees criticized certain segments of the abortion rights movement for using anti-motherhood or anti-childhood messages without recognizing motherhood as a valid choice. Interviewees emphasized the need for a more empathetic and patient approach grounded in understanding different contexts and an inclusive awareness of the diverse needs of women and people with the capacity to gestate.

### 4.4 Movement structures and sub-networks

**Figure 1:**
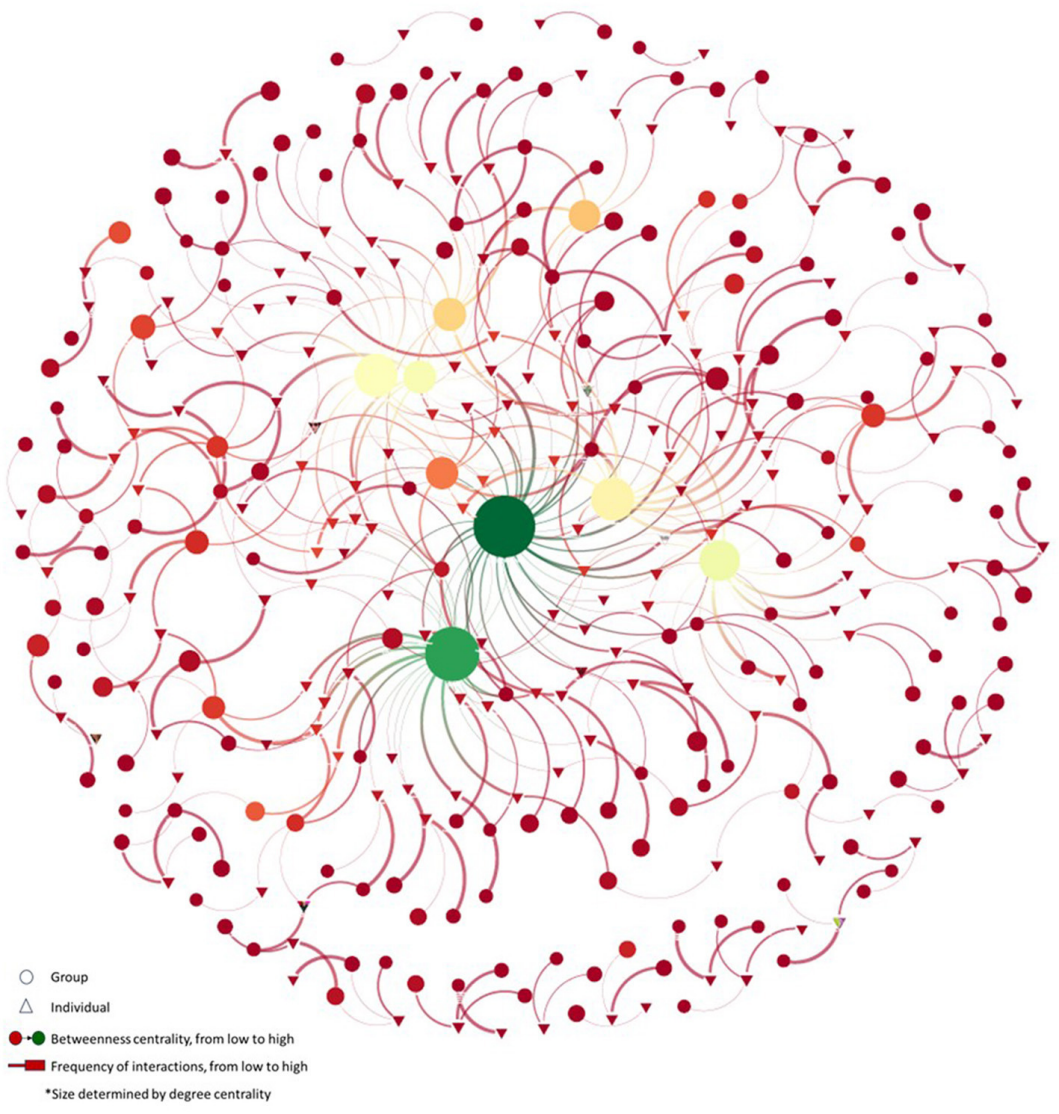
Network of the movement for the right to abortion in Mexico.

Following the typology offered by Diani (2003), using the information from 176 survey responses, the SNA characterized the movement for the right to abortion in Mexico as a “wheel” structure consisting of central nodes facilitating exchanges across the network, peripheral nodes less directly connected, and connecting nodes that bridge the entities and individuals on the periphery with the central hub^16^. The network included 350 unique elements (including formal and informal organizations and individuals) and 423 connections. Figure 1 depicts a version of the network where actors' names are concealed to protect privacy. An interactive version of the figure was shared during validation meetings, allowing attendees to explore the profile of each mapped movement actor by clicking on the nodes.

In the figure, circles represent groups, networks, collectives, and organizations, while triangles denote individual activists. Aligned with our conceptual framework that emphasizes degree and betweenness centrality, the size of actors is determined by their degree centrality, where larger sizes correspond to a greater degree of centrality. Nodes are color-graded from red to green, with the greenest shades indicating the highest coefficients for actors' betweenness centrality. The thickness of the connecting line between nodes signifies the frequency of interaction, ranging from daily to less than once a year, with thicker lines indicating more frequent interaction^17^.

It is crucial to emphasize that the mapped connections between individuals and groups stem from individual perceptions, given that the survey was responded to at the individual level, and we did not gather information representing groups' perspectives. This implies that, by survey design, we cannot assert or confirm whether mapped groups reciprocate or maintain a relationship with the individuals connected to them. Regarding the frequency of connections, only 25% (106) of the connections were reported to occur daily or weekly, while 60% (254) occurred equally or less than twice a year and 15% (63) occurred quarterly. However, it's worth highlighting that the survey was conducted during the COVID-19 pandemic, and people were adapting to remote forms of collaboration. It was discussed during the validation meetings that this indicator has likely changed. Exploring this variation in the current context could be interesting, as study participants reported that it is now easier to connect with any type of movement actor through virtual alternatives.

Visualizations and SNA centrality measures were generated using the relationship mapping software Kumu. Closeness centrality was calculated but showed no significant variations across coefficients, leading us to decide not to include it in the map. For reflection and sense-making activities (interviews and validation meetings), we concentrated on measures identified as most relevant in the literature we consulted, namely, degree and betweenness centrality. Degree centrality, as per Kumu, involves counting the number of connections an element has, while betweenness centrality measures how many times an element lies on the shortest path between two other elements.

The analysis of positionality and intermediation, conducted by calculating centrality measures, facilitated the identification of several influential organizations. It is essential to note that a high degree of centrality does not necessarily imply a connection with the broader network. Betweenness centrality addresses this by identifying elements that could bridge information within the entire network or potentially act as points of failure. As depicted in Figure 1, there was a significant overlap among nodes identified by these two centrality measures. These organizations not only acted as connectors between the central network and peripheral constellations but also served as essential brokers and amplifiers for information flow. Visually, the largest circles present the greener shades, indicating that these groups are key actors highly connected and positioned as intermediates, potentially bridging information flow across themes, identities, and geographic locations. Moreover, the prominence of identified key actors, predominantly located in the center of the network, underscores the potential for movement actors to build relationships with others through existing and positionally centered connections.

Centrality measures were additionally calculated based on respondents' affiliation, where the anonymization of movement actors was prioritized by replacing individual names with their affiliations, to measure positionality and identify relevant organizational actors. This approach ensures the privacy of individuals and provides more relevant insights for actors within the network, as they might be more familiar with the organizations than the individual names. In this exercise, seven movement actors, encompassing both formal and informal organizations, as well as formal and informal networks of organizations, emerged as significant nodes in the network map due to their degree and betweenness centrality. Details of these seven actors, including their name, geographical scope, headquarters, and the calculated measures of centrality at the group level, are provided in Table 2. The ranking of coefficients was identical when the analysis was performed at the individual and group levels – i.e., the ranking of centrality presented in Figure 1 map (individual level) matched the ranking of centrality identified at the group level (Table 2). Additionally, it is worth noting that the inclusion of specific organization names does not pose a potential ethical or safety concern, given their well-known status within the movement, as well as their public support, advocacy, and work on abortion and sexual and reproductive rights in the country and the Latin American region.

**Table 2:** Key movement actors, by scope and centrality coefficients.

**Movement actor**	**Geographic scope**	**Degree**	**Betweenness**	**Closeness**
Fondo Semillas	Formal organization headquartered in Mexico City, national reach	65	0.236	0.478
Grupo de Información en Reproducción Elegida (GIRE)	Formal organization headquartered in Mexico City, national reach	55	0.207	0.454
Fondo María	Formal organization headquartered in Mexico City, national reach	40	0.119	0.423
Red de los Derechos Sexuales y Reproductivos en México (ddeser)	National formal network	40	0.11	0.41
Católicas por el Derecho a Decidir	National formal network	37	0.127	0.424
Las Libres	Formal organization headquartered outside Mexico City, national reach	27	0.123	0.399
Marea Verde	National informal network	27	0.089	0.397

Although Figure 1 depicts a centralized structure (according to Diani's classification) and the main organizations presented in Table 2 are located at the center of the network, interviewees did not perceive the movement as entirely centralized, contrary to the interpretation of the mapping results. They emphasized that the agenda was not imposed on them by mainstream organizations located in Mexico City or other urban areas. This is corroborated by the centrality measures described in Table 2, where four of the nodes have activities outside of the capital and involve activities or organizations in more than one state, while the other three are headquartered in Mexico City and are considered mainstream groups.

Specifically, the identified formal networks of organizations are present in multiple states nationwide and represent various collectives and organizations across several states. The identified groups based outside Mexico City have coordinated multi-state efforts and have become a crucial reference point for the movement for the right to abortion in the country. Additionally, the informal network has a strong presence on social media, even across countries, enabling international visibility and the potential for collaboration and coordination to support local activities. Nevertheless, interviewees and participants in validation meetings, who represented organizations outside the central areas and smaller groups, did report logistical and administrative challenges associated with the geographic centralization of financial resources in Mexico City. These challenges included difficulties in navigating processes like fundraising and budgeting.

#### 4.4.1 The abortion accompaniment sub-network

The analysis revealed potential sub-networks and connections between the abortion movement and other social movements showcasing opportunities for collaboration and mutual support. Particularly, insights emerged during interviews, deepening our understanding of the abortion accompaniment sub-network, a crucial component within the broader movement advocating for the right to abortion in Mexico. This discovery was not evident during our initial analysis. Our iterative approach of analyzing survey data, expanding through interviews, and cross-referencing with the survey data revealed that the network for accompaniment plays a significant role in driving the overall abortion rights movement.

**Figure 2:**
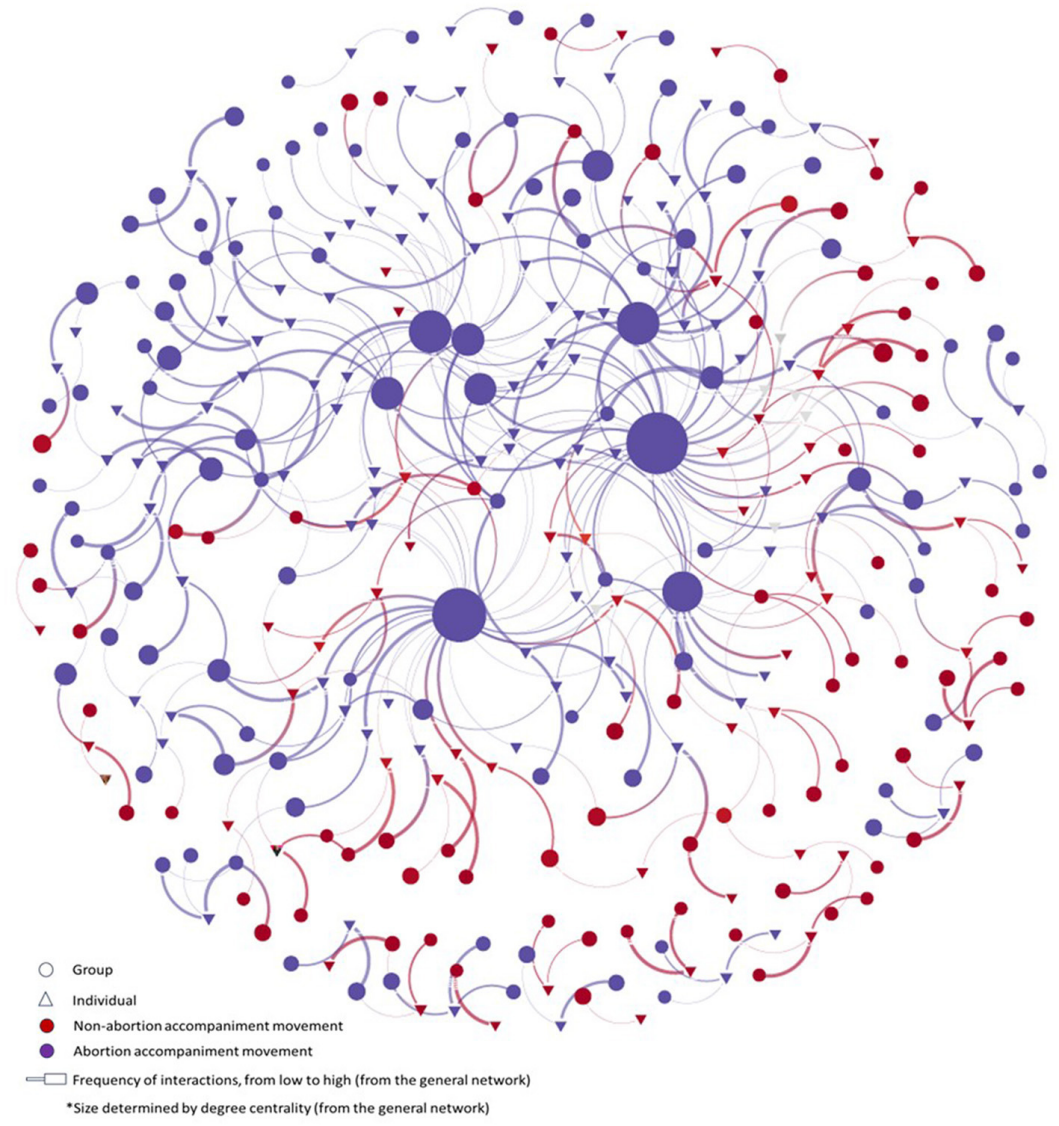
Network of the movement for the right to abortion in Mexico, by provision of accompaniment services.

While we also explored other sub-networks, such as networks of groups and activists who promote policy or legal changes, we found that the abortion accompaniment one was more significant at the moment of analysis. This could be due to data collection timing and the Supreme Court's decriminalization ruling, which probably decreased the urgency to advocate for policy changes and elevated the importance of shifting resources to providing safe abortion services. The accompaniment sub-network comprises 243 unique elements and 287 connections, representing 70% of the overall network, and follows the same “wheel” structure with a few disconnected actors. Additionally, the same movement actors from the overall abortion network were identified as the most relevant in the accompaniment sub-network in the SNA centrality analysis. Figure 2 presents the same network mapped in Figure 1, with actors playing some role within the abortion accompaniment sub-network highlighted in purple. As mentioned in a previous section, this includes those involved in direct accompaniment services, midwife services, healers, health professionals, etc.

Interviewees highlighted the solidarity and connections forged by abortion accompaniment providers – companions – who focus on supporting with financial and non-financial resources the abortion processes of women and individuals with the capacity to gestate. Interviewees also mentioned that these connections extend nationally and even internationally, enabling the accompaniment from other countries. While many companions provide support throughout any feasible week of gestation, prioritizing the goal of ensuring that no pregnant woman or person is forced to give birth, some companions could restrict their support to certain stages of pregnancy due to fear of criminalization, and some rural communities' stigmas. However, a shared theme that resonated through the interviewees' accounts is the clear emphasis on the fundamental significance of providing accompaniment, regardless of the stage of pregnancy involved. Finally, interviewees stated that while some companions prefer to remain anonymous for security reasons, others share that promoting anonymity contributes to the stigma surrounding abortion.

## 5 Discussion: identifying relational dynamics and collaboration challenges

By implementing our mapping methodology to the movement for the right to abortion in Mexico, we were able to refine our tools, process, and analysis significantly. The adaptation stage was particularly vital in addressing the case study's unique contexts, languages, and terminologies, both technically and politically. The partnership between Global Fund for Women, seasoned in data collection and analysis to support social movements worldwide, and Fondo Semillas, deeply rooted within the Mexican abortion rights movement, fostered continual discussions and validation of decisions throughout every stage of the mapping process, from case study conception to execution and analysis. While the Global Fund for Women team contributed insights gleaned from previous support to other women's and feminist movements, the Fondo Semillas team leveraged their extensive network within the movement to ensure comprehensive and inclusive participation in surveys, interviews, and validation meetings, thereby bolstering the legitimacy of findings and recommendations.

Moreover, incorporating qualitative data collection was a key in providing a more comprehensive understanding of network dynamics that quantitative data alone could not capture. The qualitative insights provided valuable directions for visualizations and facilitated the exploration of network interactions not easily measured by the survey. The case study findings and reflections provided a platform for engaging in meaningful discussions with actual movement actors, allowing us to explore whether mapped relationships accurately reflect the priorities and needs of a social movement at a given point in time.

In this section, we delve into how our methodology assists us in uncovering challenges in forming connections within the case study. A notable challenge to establishing connections was the exclusion of Indigenous peoples and people with disabilities within the movement. Several interviewees expressed concerns about the movement's limited representation of these voices and emphasized the importance of creating spaces that allow them to advocate for themselves. This concern is substantiated by the fact that representatives of these groups did not engage in the survey. This lack of participation could be attributed to our inability to reach them through direct invitations and the snowballing mechanism, their exclusion from the network, or their lack of interest in engaging with the study^18^. During validation meetings, various actors within the movement emphasized the significance of abortion companions and the demand for abortion procedures in rural and Indigenous communities and among people with disabilities, thereby dismissing the possibility of the movement lacking relevance in those contexts.

Another identified struggle was the differences in strategies often associated with generational gaps. For example, older movement members were reported to discredit younger individuals based on their preferred mobilization strategies, while a minority of young members faced criticism for promoting transphobia, influenced by narratives from established and influential feminist activists working at reputable institutions like the National Autonomous University of Mexico and the Museum of Memory and Tolerance – both institutions supposed to serve as havens for tolerance, non-discrimination, and safe spaces for vulnerable and marginalized communities, including transgender and gender non-conforming people. These observations underscored tensions within the movement, generating the perception that some actors are authoritarian and excluding other sectors from movement work.

We also identified challenges from constrained financial and non-financial resources, including safety and security resources. While larger organizations and those in the country's central regions enjoyed relatively more resource accessibility, interviewees consistently reported a persistent dearth of resources. Furthermore, the movement grappled with adversarial pressures from anti-rights groups, exerting their influence across diverse domains such as educational institutions, healthcare facilities, and the decision-making processes centered in the country's capital. And, even when organized anti-rights groups were absent, the perceived moralistic attitudes prevailing within these spheres gave rise to ongoing violent threats. Survey responses from the youngest participants prioritized allocating resources to movement actors' safety and security, which aligns with the threats and prevailing sense of insecurity experienced by some movement members.

Also, the study generated insights into how the COVID-19 pandemic disrupted various activities within the movement for the right to abortion, as it did in other sectors. Some interviewees and validation meeting participants saw the pandemic as an opportunity to geographically decentralize the movement by increasing online work and reducing the need for in-person meetings. However, they also agreed that this shift to virtual platforms reinforced the isolation of marginalized voices without adequate internet access. It was also reported that the increased demands of care work during the pandemic further impacted the participation and engagement of women, who constitute a significant portion of the movement.

In summary, the movement advocating for the right to abortion in Mexico in the analyzed period encompassed a diverse spectrum of perspectives, struggles, and network dynamics. Nonetheless, insights gleaned from the study indicated that these movement factions could find common ground in the face of shared challenges, thereby highlighting opportunities for collaboration. Among the considerations for effective collaboration is the need for adaptable financial support to respond to a dynamic environment. This support should include measures to enhance the safety, security, and overall wellbeing of movement actors. Additionally, recognizing the importance of bringing unheard voices to decision-making processes, prioritizing the engagement of informal or unincorporated organizations, such as collectives and networks, emerged as a pivotal strategy for bridging gaps and ensuring the involvement of disconnected actors. Lastly, the notion of creating intergenerational spaces for exchange and mutual learning was noted, acknowledging the value of youthful leadership and the wisdom amassed by longstanding movement participants. This approach should be underpinned by a deliberate commitment to maintaining or pursuing the intersectional perspective that seems to guide abortion work among most movement actors who participated in the case study, prioritizing the inclusion of actors from systematically excluded contexts.

## 6 Conclusion

Mapping movements can be critical to uncovering resources, coordination modes, structures, and relationships across a movement network. The mapping methodology described in this article couples a survey and semi-structured interviews to visualize and analyze movements to identify areas for action and/or resources that may enhance how the movement functions, with specific attention to less connected, less resourced, or otherwise marginalized actors. The case study process integrates insights from three strands of social movement literature, acknowledging the intricate networks within movements, the significance of relationships for resource sharing, and the usefulness of network structures. This includes understanding movement actors' presence, positionality, and capacity to support or impede the flow of information, contributing to a comprehensive understanding of movement dynamics.

Valuable insights were gained regarding resource distribution within the movement, identification of key players, movement priorities, and sub-networks, challenging preconceived notions about the impact of certain groups and activists. The chosen case study on the movement for the right to abortion in Mexico was timely, given the transformative period in the region's abortion rights landscape. Specifically, our findings have allowed for a more nuanced understanding of one of the most compelling social movements to have occupied the Mexican political space in the past decade. They also shed light on what movement actors identify as their needs and challenges, enabling the discussion of strategies to address aspects such as financing, articulation, and organizational strengthening. This fosters an environment of collective learning and reflection, promoting the sustainability of the abortion rights movement and its social change outcomes.

The network analysis approach to studying social movements has limitations that must be acknowledged. Firstly, the analysis provides only a snapshot of the movement at a specific moment, while social movements are dynamic and constantly evolving. To accurately understand the movement, multiple data collections and analyses would be necessary to keep up with its changing priorities and challenges. Secondly, accessibility can be a challenge, particularly for marginalized groups facing barriers to participation, such as limited internet access and concerns about privacy and security. However, strong local collaborations and partnerships can help address these challenges by providing targeted outreach and data collection to ensure diverse voices and perspectives are included.

Additionally, there is a risk of extracting information from social movements without giving back to the communities involved. It is crucial to be transparent, provide actionable insights, and engage in ongoing conversations and collaborations that elevate the voices of participants and overall movement actors. For instance, in this case study, we prioritized transparency through participatory processes involving movement actors in validating quantitative data. This approach ensured the discussion of potential biases, thereby addressing data and methodological gaps and bolstering the reliability of the analysis. Once key movement actors validated the appropriateness of the analysis and provided additional context through interviews, we engaged in broader collective discussions regarding the pertinence of the findings and the optimal use of the information. This collaborative approach ensured that findings were disseminated in ways that resonated with the needs and priorities of movement actors. Ultimately, the collective activities involved in the proposed mapping methodology were designed to translate into actionable measures for resource mobilization and to build bridges for collaboration within the network.

This case study presents a valuable approach to studying social movements through an intersectional feminist lens. The analysis of the movement for the right to abortion in Mexico demonstrates the effectiveness of the proposed methodology in analyzing the network structures within the movement. By gaining insights into the roles and relationships of movement actors, their priorities, and challenges, this approach can inform advocacy and activist strategies, particularly in contexts characterized by violence, oppression, and relational struggles. By understanding and addressing network dynamics, social movements can strive for meaningful change and work toward achieving shared visions and collective goals.

## Data availability statement

The raw data supporting the conclusions of this article will be made available by the authors, without undue reservation.

## Ethics statement

Ethical approval was not required for the studies involving humans because Institutional Review Board approval (IRB) was not sought. Ethical considerations were prioritized by obtaining informed consent from individuals who participated in the surveys and interviews, and no private data or identifiable information was used for the analysis, ensuring that privacy and rights are protected. The studies were conducted in accordance with the local legislation and institutional requirements. The participants provided their written informed consent to participate in this study.

## Author contributions

CL: Writing – original draft. AS: Writing – review & editing. DM: Writing – review & editing.

## Footnotes

^1^For a deeper understanding and discussion of SNA and the implication of nodes positionality and centrality coefficients calculation (see Wasserman and Faust, 1994; Freeman, 2002; Scott, 2012).

^2^Global Fund for Women is a US based feminist fund supporting social movements that build grassroots people's power to transform economic, political, and cultural systems that perpetuate gender injustice.

^3^Fondo Semillas (Mexico), Fondo de Mujeres del Sur (Argentina), Mongolian Women's Fund, Ukrainian Women's Fund, Women's Fund in Georgia, and Taso Foundation (Georgia).

^4^Fondo Semillas is a feminist fund based in Mexico that believes in the transformative power of organized girls, women, trans^*^ and intersex people.

^5^The MCAT engages multiple stakeholders in a collective assessment of the ability of a set of loosely organized and interconnected actors to collaborate to challenge the status quo and build and sustain public pressure toward their vision of the future. The tool collects information to (a) understand how stakeholders perceive capacity, (b) identify movement priorities and challenges, and (c) assess whether there is agreement about these priorities and challenges. Movement capacity is measured based on movements' grassroots base, leadership, vision and narratives, collaboration & coordination, and safety and security. The tool is publicly available upon request at: https://www.globalfundforwomen.org/apply-for-a-grant/movement-capacity-assessment-tool/.

^6^Please note that this questionnaire was adapted to examine the movement for the right to abortion in Mexico between September and December 2021.

^7^Global Fund for Women utilizes Qualtrics because of its strict privacy and security policies that comply with General Data Protection Regulation (GDPR) standards and its provision of a contact list feature that we programmed to support the snowballing mechanism.

^8^The initial raw dataset included 291 response attempts, which, after cleaning and ensuring each response had enough information to map the respondent's perception of the movement, resulted in a final sample of 225 responses.

^9^Survey participants had the opportunity to indicate up to three of their own affiliations (highlighting their main affiliation) and up to three of their most frequent relationships at the time of data collection. Respondents were also invited to specify whether their main relationships occurred within their own affiliations, indicating if someone from a certain group was mainly connecting with people within that group. Three respondents (1%) reported having some type of affiliation with Fondo Semillas, and all three also reported a relationship with Fondo Semillas. However, only one of them identified Fondo Semillas as their main affiliation. Thirty-seven respondents (16%), including the three affiliated with Fondo Semillas, reported a relationship with Fondo Semillas. This allowed us to verify that movement perceptions were not solely driven by individuals working internally at Fondo Semillas and that we were able to reach groups and activists beyond Fondo Semillas' direct network. Importantly, this doesn't imply that the reached actors were unfamiliar with Fondo Semillas or had not worked with them in the past. For example, after replacing reported relationships (individual names) with their most likely affiliations to maximize their privacy, we identified 66 relationships involving Fondo Semillas out of the 423 total mapped relationships. Further details are provided in the subsequent Results section.

^10^The complete report in Spanish is available on Fondo Semilla's webpage at: https://semillas.org.mx/pdf/ConstelacionesDerechoAlAbortoMexico.pdf.

^11^For additional details regarding the decision made by the Mexican Supreme Court to decriminalize abortion, you may refer to the article “Mexico's Supreme Court Votes to Decriminalize Abortion,” authored by Kitroeff and Lopez (2021). It can be accessed on the New York Times website through the following link: https://www.nytimes.com/2021/09/07/world/americas/mexico-supreme-court-decriminalize-abortion.html.

^12^This was an open-ended question for which the case study team could identify common topics that were later validated during interviews and validation meetings.

^13^The movement stages included in the questionnaire were derived from the Global Fund for Women's MCAT, which was developed based on data collection and discussions held across over seven movements worldwide in collaboration with local feminist funds. These definitions were included in the survey questionnaire and are also available in Appendix 1: (a) Emerging: there is widespread discontent about the status quo, a few organizations and their members are raising awareness of the issues, and many engaged in the work are not aware of or connected with each other, there is no shared movement identity; (b) Popular: discontent becomes visible through mass actions, typically triggered by a well-publicized event. Individual participants become aware of one another and start to form relationships. The movement is building a shared, collective identity and is gaining more media attention, broader public participation, and becoming a more prominent political force; (c) Formalizing: movements become more professionalized with formal organizations, paid staff, and coalition-based strategies. They have greater access to and recognition by political elites and engage in less mass action strategies, and (d) Decline: At any point in their lifecycle, movements can lose momentum due to repression or co-optation or because they have succeeded in achieving their goals.

^14^The green handkerchiefs have become the main symbol across the Latin American movement for abortion, known as Marea Verde (“green wave”) movements.

^15^Although transphobia has been a growing topic of discussion within feminist and women's movements worldwide, the case study team did not expect to find its relevance within the analyzed abortion network. As a result, there were no specific questions addressing this issue. While a few open-ended survey responses did highlight this factor, it overwhelmingly drove discussions during interviews and validation meetings.

^16^This “centralization” consideration pertains to the configuration of network interactions and is independent of geographic considerations. However, as explained later, it is noteworthy that some pivotal actors, as determined by centrality metrics, are situated in the country's capital city, where social and governmental services are also located.

^17^The survey collected various indicators, including the likelihood of sharing financial resources or confidential information, to characterize connections. In most cases, this likelihood was very high, and we did not observe significant variations across connections that could be visually represented. The survey prompted respondents to describe their main relationships, and it might have been anticipated that respondents would nominate those with whom they are very likely to engage in various activities within the movement. However, during the tool's design phase and discussions involving other movements or contexts, the case study teams considered the possibility of varying likelihoods depending on levels of distrust. Consequently, we deemed it relevant to retain these questions, even if no respondents characterized their main connections as unlikely to perform certain activities.

^18^Upon identifying accessibility challenges, such as the absence of internet access, the on-ground team took proactive measures to engage directly with movement actors. This entailed conducting targeted interviews and reinforcing the significance of survey participation through text messages or WhatsApp reminders.
