# Supplementary material for: Mapping social movements momentum: unveiling networks in the movement for the right to abortion in Mexico
Source: Front Res Metr Anal. 2024 May 24;9:1294495. doi: 10.3389/frma.2024.1294495 (PMC11157092; doi:10.3389/frma.2024.1294495)
Supplement: Supplementary file 1 [file Data_Sheet_1.docx]

# Appendix 1. Questionnaire

This survey questionnaire was designed to study the movement for the movement for the right to abortion in Mexico from September to December 2021. Contact the researchers for an updated version of the questionnaire.

## Intro. Welcome message describing the purpose of the survey, including consent language and opt-out details.

## Demographic Information:

Q1 Please, provide your name and email address.

Q2 What is your age? [List of age categories]

Q3 What is your gender? [List of gender categories]

Q4 What is the main location of your activities? [List of geographic locations]

## Affiliation and Role in the Movement:

Q5 Please select the main way in which you contribute to the movement [List of movement roles/activities]

Q6 If you are part of a sub-network, please indicate the main way in which you contribute to it [List of sub-network roles/activities]

Q7 Do you see yourself as part of this movement? [Yes/No]

- If No, the respondent is asked to explain why, share their general perception of the movement, and whether they wish to respond to the rest of the survey.

Q8 What are the main visions/priorities of the movement? [Open-ended]

Q9 Are you affiliated with an organization formal/informal? [Yes/No]

- If yes, the respondent is asked to provide the name and identify the type of organization

Q10 Please indicate the capacity area/dimension that best describes your individual or collective collaboration with the movement: [List of MCAT capacity areas. See Q12-Q16]

Q10 If more than one aspect was selected, please rank them.

Q11 What is the most important outcome the movement is trying to achieve? [Open-ended]

## Movement Capacity Assessment (MCAT):

*Q12-Q16 Please rate your level of agreement about different aspects of the movement. For each item, please select your level of agreement from 1 being “Strongly Disagree” to 5 being “Strongly Agree.”*

| **Capacity Area/Dimension Agreement** | |
| --- | --- |
| **1. Grassroots Base** | |
| 1.1 Members of the movement effectively bring individuals directly affected by the issues together to demand change |  |
| 1.2 Individuals who are most affected by the issues are speaking for themselves and advocating for change |  |
| 1.3 Members of the movement effectively engage the general public to join the movement |  |
| 1.4 Members of the movement have mobilized a large number of the population multiple times to take action to demand change |  |
| 1.5 Members of the movement have increased attention from power holders through collective action |  |
| **2. Leadership** | |
| 2.1 Leadership listens and responds to the needs of the communities affected by the issue |  |
| 2.2 Leadership creates ways for new leaders to emerge and grow |  |
| 2.3 Young members of the movement take on leadership roles |  |
| 2.4 Leadership is effective in identifying and responding to opportunities |  |
| **3. Vision and Narratives** | |
| 3.1 Members of the movement share a clear vision of what we stand for and what kind of world we are trying to create |  |
| 3.2 Members of the movement are effective in communicating the vision with the general public |  |
| 3.3 The strategies our movement prioritizes today will lead to achieving our vision for the future |  |
| **4. Collaboration & Coordination** | |
| 4.1 Members of the movement come together to develop a joint strategy |  |
| 4.2 Members of the movement respond quickly in a coordinated way when opportunities arise |  |
| 4.3 There are recognized organizations or coalitions that support members to communicate and coordinate effectively |  |
| 4.4 Members of the movement discuss successes and challenges in an open and honest way |  |
| 4.5 Members of the movement use decision-making processes that align with the vision and values of the movement |  |
| 4.6 Members of the movement can disagree and still work together toward the shared vision |  |
| **5. Safety & Security** | |
| 5.1 Members of the movement come together to identify risks and develop plans for digital security |  |
| 5.2 Members of the movement come together to identify risks and develop plans for physical safety |  |
| 5.3 There are support networks to provide immediate response to address violence against members of the movement |  |
| 5.4 There are sufficient opportunities for members of the movement to take care of their mental, emotional, and physical health |  |

Q17 Among the dimensions above, which is the movement's highest priority (most urgent now)?

- The respondent is asked to identify the most urgent sub-capacity area/sub-dimension of the selected priority

Q18 How would you strengthen the identified priority? [Open-ended]

Q19 Social movements exist in different states in different periods of time. This is based on the current environment, the tactics or actions being used, the size and diversity of the movement, and the ways individuals and organizations are working or not working together.  What state do you believe the movement is in currently?

| **State** | **Description/characteristics** |
| --- | --- |
| Emerging | There is widespread discontent about the status quo, a few organizations and their members are raising awareness of the issues, and many engaged in the work are not aware of or connected with each other, there is no shared movement identity. |
| Popular | Discontent becomes visible through mass actions, typically triggered by a well-publicized event. Individual participants become aware of one another and start to form relationships. The movement is building a shared, collective identity and is gaining more media attention, broader public participation, and becomes a more prominent political force. |
| Formalizing | Movements become more professionalized with formal organizations, paid staff and coalition-based strategies. They have greater access to and recognition by political elites and engage in less mass action strategies. |
| Decline | At any point in their lifecycle, movements can lose momentum due to repression or co-optation, or because they have succeeded in achieving their goals. |

Q20. Please mention three risks or challenges affecting the movement [Open-ended]

## Network Characteristics:

Q21 Please list up to 3 of your most important collaborations, relationships and/or affiliations. This may include individuals, groups, organizations, networks, coalitions and campaigns. Please, do not include your main affiliation.

Q21.1 Is *Relationship* an individual or a group?

- If respondent selects “individual,” then they are asked whether the Relationship is affiliated with a group

Q21.2 Which of the following best describes your most recent interactions with *Relationship*? [List of types of interactions]

Q21.3 How frequently did you interact with *Relationship*? [List of frequencies]

Q21.4 What has been the main success or gain because of your relationship with *Relationship*? [List of gains]

Q21.5 In a scale from “1 - Definitely will not” to “5 - Definitely will”, how likely would you be to do the following with *Relationship*?

- Share financial resources
- Plan and implement joint actions
- Share confidential or sensitive information
- Recommend or connect to other movement actors

Q22. Would you like to provide the contact information of the Relationships you mentioned in the previous section? If so, please enter their email addresses
 
Q23. Would you like to provide feedback on your experience responding to this survey? [Open-ended]
