# Supplementary material for: Mapping social movements momentum: unveiling networks in the movement for the right to abortion in Mexico
Source: Front Res Metr Anal. 2024 May 24;9:1294495. doi: 10.3389/frma.2024.1294495 (PMC11157092; doi:10.3389/frma.2024.1294495)
Supplement: Supplementary file 2 [file Data_Sheet_2.docx]

# Appendix 2. Invitation Email and Consent Language

Sender: Fondo Semillas

Subject: Semillas is inviting you to participate in a survey assessing the movement for the right to abortion in Mexico

Hello!

We hope this message finds you well and healthy.

Fondo Semillas is conducting a study to understand the movement for the right to abortion in Mexico better. To carry out an appropriate analysis of this movement, we have partnered with the Global Fund for Women (GFW) evaluation team, who will administer the application of a survey, which is one of the tools that will feed the study.

We would very much like you to participate in this survey, as it will allow us to have a broader picture of the movement, however, your participation is completely voluntary. Likewise, the form will ask you to give references to other people who participate in the movement for abortion rights in Mexico in order to be able to send them the survey. It will be open until November 5.

By participating in this survey, you agree that the information provided (names, emails, etc.) is used for the purposes mentioned above. This information will be protected by Fondo Semillas. Please consult GFW's Privacy Policy [link to Policy] and Fondo Semillas's Privacy Policy [link to Policy] if you want to understand how we will protect the information you share with us.

If you have any questions about this project, before or after participating in the survey, please write to mcat3@semillas.org.mx

We thank you in advance for your interest in participating.

Receive a warm greeting.

[URL: “Click to respond survey”]

[URL: “Copy and paste this link in your browser”]

[URL: “Click if you wish to opt out from the study or receiving these emails”]
